# Implementing a clinical-educator curriculum to enrich internal medicine residents’ teaching capacity

**DOI:** 10.1186/s12909-019-1888-0

**Published:** 2019-12-11

**Authors:** Yacob Habboush, Alexis Stoner, Claribel Torres, Sary Beidas

**Affiliations:** 1 Department of Internal Medicine, Orange Park Medical Center, 2001 Kingsley Avenue, Orange Park, 32073 FL USA; 2 Department of Preventive Medicine and Public Health, Edward Via College of Osteopathic Medicine, Spartanburg, SC USA; 3 Department of Research and Sponsored Programs, Jacksonville University, Jacksonville, FL USA

**Keywords:** Clinical educator, Graduate medical education, Residents, Medical students

## Abstract

**Introduction:**

Physicians-in-training (residents) are typically the primary educators for medical students during clinical clerkships. However, residents are not formally trained to teach or to assess their teaching. The aim of this study was to assess the implementation of a clinical educator rotation aimed at developing residents’ competencies related to clinical teaching.

**Methods:**

A mixed-methods approach was used to develop and assess the clinical educator rotation at a teaching community hospital. Internal medicine residents who participated in the rotation and consented to the research were assigned to the clinical educator trainee (CET) group, the remaining residents were assigned to the control group. Osteopathic medical students rotating in the medicine service line were invited to participate. The study used descriptive and qualitative analyses to measure primary and secondary outcomes.

**Results:**

The primary outcome measure showed a positive change in resident knowledge, skills and behaviors in communication, reflection, feedback, precepting, and facilitation. Medical student perceptions of resident teaching skills confirmed the observed changes in CETs. Some CETs continued to practice and build their capacity for teaching after completing the rotation. Qualitatively, we derived four common themes among the data; communication, professional engagement, practice-based learning, and systems-based learning.

**Conclusion:**

Resident teaching capacity was enriched after completing the clinical educator rotation. Other benefits included: enhanced patient communication and education, increased resident confidence, personal satisfaction with training, work life-balance and enhanced career satisfaction. Future research should focus on curricular content, faculty development, and delivery assessment. In addition, research efforts should identify appropriate emerging technologies to include in the curriculum for enhancing teaching capacity.

## Introduction

In response to projected physician shortages in the United States,[1] there has been a rapid expansion of graduate medical education (GME) programs. In 2017–2018, 620 new GME programs were launched [2]. Alongside this rapid expansion in GME programs, are extensive changes in technology, healthcare, and education delivery methods.

Physicians-in-training (residents) are typically the frontline primary educators for medical students during clinical clerkships. Even after residency, many residents continue to engage as educators becoming clinical faculty in medical schools and teaching hospitals. Residents are estimated to contribute approximately two-thirds of medical student education during the clinical third and fourth years [3]. After graduating from medical school, there is an implicit expectation that medical students assume the role of educators as they transition into residency. However, most medical academic curricula are crowded and do not allow for the time to include developing residents’ teaching competencies [4–6]. As a result, residents are left with the challenging task of teaching without having the training or skills to be effective educators. As GME continues to expand, programs will need to invest in developing or acquiring faculty with a skillset to promote positive resident educational experiences leading to successful future clinical careers [7–10]. One method to do so is to institute a clinical-educator track geared towards a resident audience [11–13].

In fulfilling this approach, we developed and initiated a “clinical-educator” rotation for post-graduate-year two (PGY-2) and three (PGY-3) residents in Internal Medicine (IM) The rotation introduces learning theory mixed with pragmatic skill development aided by the use of digital technologies to leverage residents’ teaching capacity within our institution [14]. The primary objective of this study was to assess the impact of the clinical-educator rotation on residents’ competencies related to clinical teaching. Secondary objectives were to determine medical student perceptions in reference to observed changes in residents’ teaching as measured by communication, practice-based learning, professional engagement, and systems-based learning.

## Material and methods

A prospective, exploratory descriptive study was conducted with a concurrent triangulation design to collect both qualitative and quantitative data from three stakeholder groups: medical students, residents and clinical faculty. This study was approved by the Edward Via College of Osteopathic Medicine, Institutional Review Board.

### Study population

A purposeful sampling technique was employed to recruit residents at a community teaching hospital in Orange Park, Florida. All PGY 1/2/3 residents (*n* = 30) were invited to participate in the study after an introductory presentation. Because the rotation was an elective rotation limited to PGY 2/3 residents, we selectively assigned all the PGY I residents (*n* = 10) to the control group. Residents (PGY 2/3) who selected to rotate in the clinical educator rotation were assigned to the test group, clinical-educator trainees (CET). All PGY 2/3 residents who chose not to participate as a CET, along with PGY-1 residents, were assigned to the control group. Using a similar purposive sampling approach, all third year osteopathic medical students (*n* = 20) rotating with IM residents were invited to participate in the study. All participants in the study were consented.

### Clinical educator rotation

The curriculum was developed by drawing from the contemporary clinical-educator and faculty development literature based on participating faculty’s experience and training in faculty development [11, 15]. Traditional clinical faculty development topics such as precepting, feedback, learning theories, lecturing, and small group facilitation were delivered in weeks one and two. In addition, we added contemporary topics selected from Gonzalo et al’s, report on changing clinical faculty development needs to include: change management, patient-centered medical care, electronic health records (EHR), complexity, learning healthcare systems, and digital technologies [16]. The contemporary topics were designed to be delivered as elective modules, in weeks three and four, based on learners’ interest and needs identified with faculty collaboration [16]. In brief, the hybrid model was built on a two to four-week rotation with an emphasis on communication and practice-based learning delivered in the first 2 weeks in eight mandatory modules. In weeks three and four, residents selected up to two additional modules per week. These latter modules exposed the residents to the domains of professional engagement, and systems-based learning (Table 1). The rotation director delivered the eight modules in the first 2 weeks face-to-face and included a variety of exercises that promote dialogue, reflection, problem-solving and hands-on experience to anchor and promote the transition from theory to practice. Whereas weeks three and four elective modules were designed to be delivered either face-to-face or remotely (based on available faculty expertise) using video and supervised locally by the rotation director.

**Table 1:** Modules for the Clinical-Educator Rotation

Time frame	Category	Modules
Week 1	Communication Science^a^	· Principles of Adult Learning & Definitions · Reflective Practice / Journaling · Feedback · Electronic Health Records/Quality Documentation
Week 2	Practice-Based Learning^a^	· Precepting Skills · RIME · Mentoring · Small Group Meetings / Skills
Week 3	Systems-Based Learning	· Relationship-centered communication · Problem learner · Difficult patient · Presentation Skills using digital tools · Managing Change & Complexity · Social Media in Healthcare · Quality Improvement · Social Determinants of Health · Time Management
Week 4	Professional Engagement	· Leadership / Teams · Negotiation / Problem Solving · Patient-Centered Medical Home · Population Health · Precision Medicine · Value Healthcare · Innovation / Data & Measurement · Health Systems

RIME: Reporter, interpreter, manager, educator

^a^Week 1 & 2 modules are mandatory

The CET and control groups were involved in the usual daily teaching activities of the residency program by leading daily morning huddles, morning topic discussions, precepting, evaluating clinical notes, providing feedback, and facilitation during various topic presentations. In addition, CETs participated in weekly bedside teaching sessions for medical students. For the bedside teaching experience, we elected to identify differences at the beginning and end of rotation for each CET. Prior to bedside teaching, each CET was oriented to the objectives and format of the bedside teaching session with emphasis on pre-session student orientation to clarify objectives and respond to students’ questions. After the bedside session, the group met to debrief their reflections-on-learning. Residents from the control group did not participate in bedside teaching because of potential unfairness to residents who may be put on the spot in the presence of patients and students.

### Data collection

A total of five key assessments, designed in-house, were utilized to answer the research questions listed in Table 2. Four of the key assessments, communication, systems-based learning, professional engagement, and practice-based learning, aligned with the basic constructs that framed the study. Both the control and the CET groups completed these assessments. The fifth assessment was a summative pre/post assessment, open-ended questionnaire, to help identify if the program met the objectives. All medical students and residents completed the pre-assessment; whereas the post-assessment was completed by the CET group only. Towards the end of the study, we conducted three focus group discussions for the medical students, residents, and faculty. Table 3 demonstrates the matrix of the assessment methods. The key constructs and assessments are described below:

**Table 2:** Research objectives and questions

Primary Objective and Questions	Does the Clinical Educator rotation directly improves resident competencies related to clinical teaching and professional growth? A. *Is there a difference in how CET communicate with stakeholders when compared with the control group?* B. *Is there a difference, as compared to the control group, in how CET improve practice-based learning?* C. *Is there a difference in levels of professional engagement between CET and the control group?* D. *Is there a difference, as compared to the control group, in how CET demonstrate systems-based learning?* E. *To what extent program objectives are met from the perspective of the clinical educator trainees?*
Secondary Objective and Question	Assess medical students’ perception of how do clinical educator trainees use and apply communication, practice-based learning, professional engagement, and system-based learning?

*CET* clinical educator trainee

**Table 3:** Matrix of assessment methods

Assessment Tools	Triangulation Methods					Themes
	Observation	Video	Journaling	EHR	Focus Group	
Huddle	Faculty	CET/C	–	CET/C	MS/R	Communication
Reflection	Faculty	CET	CET	–	MS/R	Practice-based learning
Presentation	Faculty	CET/C	–	–	MS/R	Professional Engagement
QNOTE	Faculty	–	–	CET/C	–	Systems-based learning
Pre/Post- Surveys	–	–	CET/C	–	–	All

*C* Controls, *CET* Clinical-educator trainees, *EHR* Electronic health records, *MS* Medical students, *R* Residents

#### Communication

This construct targets verbal, written, and digital communication standards with the purpose of facilitating effective communication behaviors/patterns between all stakeholders. The stakeholders included students, residents, faculty, staff, patients/families. Communication was assessed using the morning huddle survey, (an eight question likert scale survey ranging from strongly disagree to strongly agree), during the morning huddle where trainees briefly reviewed their scheduled patients. In addition, we used different strategies to assess resident communication during the rotation using observation, video, reflection and review of EHR notes.

#### Practice-based learning

The purpose of engaging residents in reflective practice was to develop residents’ capacity for life-long learning. The second assessment was therefore, reflective journaling. Each resident practiced daily journaling using Mezirows’ reflective levels [17] for transformative learning to frame their reflective discussions followed by one-on-one sessions with a faculty member to anchor their skills in reflection. In addition, weekly journal narratives were assessed via a rubric by three faculty members. The reflection rubric consisted of 4-point likert scale ranging from below expectations to outstanding to assess two components, content and personal growth.

#### Professional engagement

This construct targets the domain of professionalism practiced by the resident during interactions with stakeholders in the context of cultural diversity and transnational competence during presentations. The third assessment was presentation skills that measured if the CET demonstrated mastery of professional competencies such as the practice of empathy, cultural humility in a culturally diverse context, mastery of knowledge content, role modeling, and appropriately (voice, tone, body language, etc.) responding to learner or patient difficult interactions. CET participants were given two choices of presentations: prepare a 15–20 min presentation around evidence-based physical examination and deliver the presentation in a small group meeting or facilitate a small group teaching session, (60–75 min), on an inpatient clinical case. A presentation rubric, 4-point likert scale, was used to assess trainees presentations ranging from below expectations to outstanding.

#### Systems-based learning

This construct targets documentation processes in the EHR as a surrogate for systems-based learning, using QNOTE, [18] a validated electronic evaluation tool used to assess clinical notes for quality by generating a quantitative score for clinical notes quality. Both resident groups completed multiple QNOTE evaluations for the same peers and provided peer-to-peer feedback regarding gaps in documentation and opportunities for improvement. QNOTE also enabled the CET group to identify the progression of residents and categorize them using the RIME (reporter, interpreter, manager, educator) model, Table 4 further explains the RIME model.

**Table 4:** Explanation of the RIME Model

RIME Model	Function
Reporter	Gather and report data
Interpreter	Interprets information, applies medical knowledge, weighs evidence
Manager	Organize and manage information and resources, prioritize differential diagnoses with respect to the evidence, suggest appropriate considerations for plan o f care
Educator	Articulate what is known, determine what needs to be known, convey medical knowledge in understandable terms to patients and colleagues

#### Program outcomes

A 7-item pre-and an 8-item post semi-structured questionnaire was designed in-house to determine if the program met its objectives. In addition to collecting data from the control and CET groups, this study aimed to triangulate its findings by capturing medical student perceptions of the clinical educator rotation as measured by communication, practice-based learning, professional engagement, and systems-based learning. Towards the end of the study period we conducted three focus groups for medical students, CETs, and faculty to assess program outcomes.

### Data analysis

Data from questionnaires, surveys, audio files, and video files were collected, summarized, and aggregated per group, CET vs. control, using descriptive statistics and qualitative content analysis. Two investigators transcribed and coded the data and ensured appropriate assignment of codes. These codes were reviewed independently by two other faculty members experienced in qualitative analysis to ensure intercoder agreement. We identified recurrent and/or emerging themes from responses in an attempt to further our understanding of how the curriculum was meeting its objectives. MAXQDA 2018.2 was used for the qualitative analysis of the data. Inferential statistics were not appropriate in this study because of the limited sample size and the descriptive nature of the study. Assessments completed by faculty members were quantified in order to establish inter-rater reliability and validity. Moreover, quantitative data were analyzed descriptively in order to establish patterns in responses. We used various strategies to strengthen the rigor of the study by assessing the credibility, dependability, confirmability, and transferability of the outcomes [19].

## Results

There was an overall improvement in CET’s knowledge, skills, attitude, and behavior in relation to the domains of communication, practice-based learning, professional engagement, and systems-based learning. Of the 10 residents who enrolled in the CET group, 8 completed the rotation. Data saturation was observed by the sixth trainee after which no new themes emerged. In addition, we observed that stakeholder (students, residents, and faculty) comments were similar suggesting triangulation of data. Persistence of changes, post CET rotation, were less pronounced for PGY-2 CETs. We observed consistent use of new behaviors and skills in 4/8 CETs. The primary outcome demonstrated a behavioral change towards embracing and repetitively demonstrating use of the theoretical frameworks in support of a learner-centered approach to teaching. Medical student perceptions confirmed the observed behavioral and skill changes described in the CET group.

**Fig. 1:**
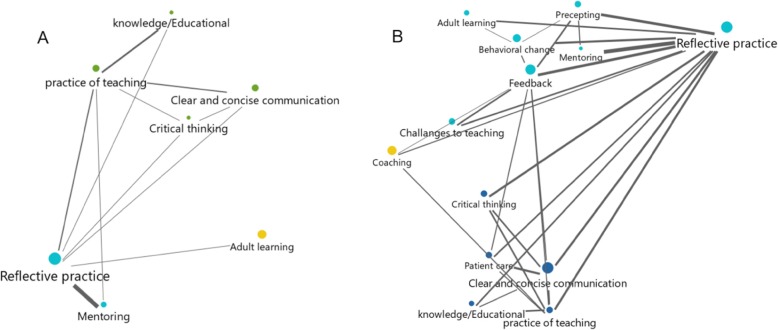
CET group: Visual showing interrelationship of codes from pre- and post- clinical-educator rotation assessments: **a**: pre-clinical educator rotation, **b**: post-clinical educator rotation. Each colored circle represents a code. Distances between the codes identifies codes that are mentioned together (overlap) in text segments; grouped by color. Connecting lines between the codes and their width reveals the frequency of co-occurrence of codes (for example: a thick line means that the connecting two codes were mentioned together frequently and often). Comparison of graphs in **a** & **b** shows evolution of CETs interpretation of clinical teaching before and after completion of the rotation. In graphic **a**, Reflective Practice and Mentoring are the primary co-occurring codes whereas in graphic **b**, co-occurring codes have significantly increased in number and interactivity

The most frequent three codes in the CET group prior to the initiation of the CET rotation were “practice of teaching” (11.8%), “critical thinking” (9.7%), and “reflective practice” (8.1%). In the control group, the most frequent three code prior to the initiation of the rotation were “practice of teaching” (14.9%), “reflective practice” (10.2%), and “challenges to teaching” (8.4%). In contrast, medical students’ top three codes were “reflective practice” (22.1%), “practice of teaching” (7.2%) and “education” (7.1%). Refer to Table 5, for examples of codes and correlating quotes. To identify relationships between the codes, a network map, based on the pre/post-survey and focus group interviews, showed a robust increase in post-rotation interconnectivity and proximity of codes to the practice of reflection. In both pre and post assessments, mentoring showed similar connectivity and proximity to reflective practice, see Fig. 1.

**Table 5:** Themes and codes. Codes are arranged in descending order according to frequency for all groups

Themes	Codes	Examples
Communication	Bedside teaching	“they did an excellent job of showing us what we haven’t learned before, like how to properly do a joint exam”
	Clear and concise communication	“her communication is super clear, we understand what we are doing and why we are doing it”
	Coaching	“She helps to lead us if we are going to stray from the point”
	Collaborative	“I know if the residents engage with us I like that cause I got to work with the residents that I wouldn’t otherwise got the chance to work with”
	Evaluation	“you are not worried as far as they are affecting you evaluation so you are taking the feedback and it is more open and easy going”
	Feedback	“I think I am better at giving feedback now that I have practiced multiple times and have received feedback on my feedback”
	Leadership	“it made me a better leader as well as more compassionate”
	Mentoring	“Mentoring is not easy either you have to focus and be understanding of a lot of things as it is not a one dimensional process”
	Planning	“It has created structure in how to approach bedside teaching”
	Questioning types	“Use questions which help them do analysis, synthesis and to increase their comprehension”
Practice-based learning	Efficiency	“he was well acquainted with him and his case, therefore the flow was very smooth”
	Motivation	“The role of a facilitator in the group by being involved and setting an example so that it motivated others as well”
	Objectives oriented	“I liked how the first day we went through our values and objectives, then began to formulate what we found to be important to us and whether we are meeting and exhibiting those values or not”
	Reflective practice	“Being more self-aware of habits and being able to sit back and think about what we are doing and why we are doing it”
	RIME	“The curriculum can help residents at different levels”
	Time management	“They are thorough and deliver information in an appropriate and timely manner”
Professional engagement	Challenges	“To identify or own premiered notions or biases”
	Cultural awareness	“Transnational competence: intricate, difficult to put into action effectively unless practiced”
	Independent practice	“You have to develop emotional intelligence, and be cognizance to treat patients as people and not numbers”
	Patient care	“it definitely improved patient care and safety”
	Practice of teaching	“the trainee did a great job keeping us focused on one subject/system/topic at a time and seemed to guide us when needed”
	Precepting	“when went to see patients he actually came with me and assessed how I did my HP. He observed me directly and when we finished the encounter he gave me feedback which was very helpful to have and kind of mentoring one-on-one”
	Professionalism	“Trainees engage in professional engagement by speaking to students properly”
	Responsibility for education	“[She] is also good at assigning patients that are good learning patients if you have seen like 3 MIs in a week, she assigns me a patient with gastritis so it’s always something new so you are not constantly seeing the same patients”
Systems-based learning	Critical thinking	“Residents innately use their own personal strategies to go about decision making”
	Evidence-based medicine	“Residents innately use their own personal strategies to go about decision making”
	Information retrieval	“We can use resources like images that was helpful and labs which was nice”
	Knowledge/ education	“Helps define reading for you that really high-yield”
	Organizational	“very organized in teaching us and for sure he was a great teacher in those three sessions”
	Technology use	“video readings helped identify areas in which I did not realize that I was appearing a certain way, and gave me concrete evidence of what I need to change”

Eight (27%) IM residents enrolled as CETs and the remaining 22 (73%) residents participated as controls. The CET group included 2 PGY-2 and 6 PGY-3 residents. A total of 17/20 (85%) medical students enrolled in the study. Eight CETs (100%) completed at least 2 weeks clinical-educator rotation with three residents completing 3 weeks. Among the residents who elected to rotate for 3 weeks, one resident completed the population health module and another the patient-centered medical home (PCMH) module. Deliverables for these modules included a Grand Rounds presented by the resident on the topic, the second resident delivered a detailed document describing implementation requirements for a PCMH practice in the resident outpatient clinic.

### Qualitative

#### Communication

The most common codes derived were ‘clear and concise communication’ followed by ‘feedback’, and ‘coaching’, see Table 4.

##### Faculty comments

From the focus group meeting, faculty commented that the CETs developed new understanding in relation to professionalism, patient care, teaching process, and planning. Communication between CET and stakeholders, including patients, medical students, staff, and faculty, was noted to be clear and organized when compared to the control group. CETs made use of verbal and non-verbal cues. Residents learned how to communicate effectively with all stakeholders to plan educational sessions.

*“During the [morning discussion] article there was more discussion compared to before.”*

*“ So, he is now involving the residents to be part of the conversation rather than him just talking so he will ask more questions and listen to them.”*

##### Evaluation tools

Using the morning huddle survey, the mean values for all questions combined were similar in both the CET and control groups with a mean of 4 correlating to ‘agree’ on the 5-point likert scale indicating a similar improvement in both groups.

##### Residents

From post-survey and focus group discussion, the residents reported that CETs showed consistency in ensuring knowledge transfer to learners.

*“Facilitating the morning group and feeling/sensing that I was listening and being listened to. I felt effective and that the whole group participated.”*

The CET group also used their communication skills to provide constructive feedback, coach learners, and ask questions at different levels of Bloom’s Taxonomy [20]. At the beginning of the clinical-educator rotation, CETs anticipated communication skills as a major challenge which was not the case after the rotation.

*“Communication skills were challenging, now I know how to effectively communicate our thought process.”*

*“...I think I am better at giving feedback now that I have practiced multiple times and have received feedback on my feedback. I feel more comfortable with it.”*

##### Students

From the focus group meeting, medical students indicated an agreement with the CETs’ improvement of communication between stakeholders. In comparison, controls were much less likely to communicate with learners through pre-planning or clarifying learning objectives when leading an educational activity.

*“...it is interesting when he first started teaching us he was kind of timid and disorganized. I gained a lot from the cases [patient care] working with him but he made a lot of improvement just from the second time he was with us as a CET. Toward the end he was very proficient very organized in teaching us.”*

### Practice-based learning

The most common codes derived were ‘reflective practice’, ‘objective oriented’, and ‘efficiency’. See Table 5.

#### Faculty comments

From the focus group meeting, faculty commented that the CET group demonstrated effective use of reflective skills and were able to provide quality feedback to learners. Their questioning styles changed based on Bloom’s taxonomy of questions and teaching was more learner-oriented. Although CETs practiced verbal and written reflection, they struggled to align their reflections according to Mezirow’s hierarchies for reflective practice.

*“So before he started the rotation he was more of a talker where he would share the knowledge he knows and he will keep going on regarding what he knows but after doing the rotation and during it he learned the skills and abilities of how can he get most out of the learners where he has now adapted the roll of a teacher and not just somebody that gives a lecture.”*

#### Evaluation tools

Based on the Reflective Journaling Rubric, most reflective practices ranged between affective through judgmental reflectivity according to Mezirow’s levels. Occasionally, a CET practiced reflection at the conceptual, psychic, or theoretical levels. The content and personal growth based on the Reflective Journaling Rubric were rated as 9% “basic”, 50% “proficient”, and 41% “outstanding”, in the CET group.

#### Residents

From the post-survey, the CETs found reflective practice to be an effective tool to help them understand their role as an educator and enhance their practice-based learning.

*“Reflective practice helped me recognize the caveats and gaps in my practice and interactions and taught me how to find remedies by self-reflection”*

*“One big part of the clinical-educator, there is actually a big mindfulness component to it so there is daily journaling and reflection in that aspect.”*

#### Students

During the focus group meeting, medical students commented that reflection practices demonstrated by the CETs were consistent and deliberate. Similar comments were echoed during resident and faculty focus group meeting.

*“Reflection is a sort of personal feedback for me where I can sit back and figure out where I could have been better; and next time, I will try to implement that and that is what [the CET] successfully did”*

#### Professional engagement

The most common codes derived from qualitative data analysis were ‘practice of teaching’, ‘patient care’, and ‘precepting’. See Table 5.

#### Faculty comments

From the focus group meeting, the faculty commented that the participants in the CET group had improved their professional interactions with their patients, colleagues, and medical students. This was evident through deliberately addressing patients and learners by name, actively organizing and planning teaching activities, and providing feedback in a facilitative and non-judgmental manner.

*“[Another CET] gave me feedback after I gave a lecture. She video taped it, as an attending you never get this opportunity where you get feedback from peers or from other attendings or from anybody. So that was helpful because by doing this rotation she developed the skills on how to give feedback without hesitation no matter if its a peer, attending, or student.”*

#### Evaluation tools

Using the presentation rubric, the CETs and controls showed similar improvement in presentation skills with a mean value of 3 correlating to ‘proficient’ in the 4-point Likert scale.

#### Residents

From post-survey and focus group discussion, the CETs demonstrated their understanding of concepts of adult learning theories, group dynamics, personal values, personal learning inventory and reflection.

*“Explaining my expectations to new students, sometimes we work together only for a couple of weeks, understanding their expectations as many medical students are too new to the clinical setting.”*

*“It is difficult to evaluate somebody and be able to add to that picture unless you are paying good attention and you are following them along as now I am able to look at the picture and see what I can bring more to the table as my perception changes you are not there to be in the room or be a part of it and say yes to what has been but your job is to make sure that how this process is taking place and where it is going and if we are going together or not.”*

#### Students

From focus group discussion, the medical students stated that CETs demonstrated a higher level of professionalism when interacting with learners. There were noticeable changes in CET behavior that medical students recognized as a different professional behavior when compared to controls.

*“[CETs] let me be a reporter and take charge and when I present the patient to the attending, they didn't interrupt me and let me do my job as a reporter”*

*“[CETs] were very professional, they called me by name, rather than ‘medical student,’ everyone calls me medical student, but they used my name. They were attentive, listen to us, and keep eye contact.”*

### Systems-based learning

The most common codes derived from qualitative data analysis were ‘knowledge/education’, ‘critical thinking’, ‘technology use in teaching’. See Table 5.

#### Evaluation tools

CETs were able to demonstrate proficiency in systems-based learning through the use of QNOTE. CETs were more inclined to identify gaps in notes with an average QNOTE score of 80.6, while controls were more inclined to give a higher score to the same clinical notes with an average score of 91.2, indicating a lack in identifying gaps in EHR note quality.

#### Residents

From post-survey and focus group discussion, CETs were able to use their newly acquired skills in different clinical settings including precepting in the outpatient clinic and bedside coaching in the inpatient service. By acting as team leaders, CETs engaged a variety of hospital systems and utilized available resources to create and enhance learning opportunities.

*“Implementation of a variety of teaching strategies appropriate to learners, engage in critical thinking and create opportunities to do so, using information technology to support the learning process, role model.”*

*“...my opportunity to look at myself from a different point of view. Looking forward to discover what are the challenges that a teacher faces while trying to meet the needs of different people who may be very different from each other in the way they learn, yet have the same objective.”*

CETs were also able to master the use of QNOTE to assist learners in identifying their gaps in clinical notes.

*“ I also had another CET and she went over my notes using QNOTE and I noticed a lot of errors so now I am looking at everything deeper.”*

#### Students

From the focus group discussion, medical students commented that CETs’ behavior change was evident during bedside teaching sessions where their actions manifested in a patient and learner-centered approach. Patients were enthusiastic to participate in the teaching session as it provided them with a deeper insight about their case. Furthermore, medical students had a unique opportunity to recall their theoretical knowledge and reflect on observed practices of clinical care to help them transition from theory to the practice of medicine.

*“When going through review of systems and physical exam, systems-based learning allows the student to compartmentalize the teaching and ensuring all aspects of patient care/differential diagnosis are addressed. At the same time, providing a method to draw from, to develop a "bigger picture" mentality with patient care.”*

In addition to collecting data on these four constructs, we aimed to assess if the clinical educator rotation met its objectives from the perspective of students, residents, and faculty. Faculty and study participants observed significant behavioral changes in the CET group after the completion of the rotation.

“*In my opinion I think that this was a really good effort. I have seen significant changes, and these changes are lifelong. It’s like you developed the muscles and you keep working and those that learn and retain it if they practice it.”*

The most noticeable behavioral changes included the ability to conduct well-structured, concise, and focused feedback to learners. For example, when precepting or leading small groups, CETs used a mix of higher order questions that engaged the learners in analysis and evaluation rather than the predominant use of low order questions such as knowledge or comprehension based. CETs provided learners with space to work through problem solving and focus on their clinical reasoning skills.

*“One thing about CET, he has always been very inquisitive and always asks good questions. He is always thinking deeper and thinking; well, what would you do in this scenario”*

Other behavioral changes that were acquired and used by CETs included: facilitating skills, concise and clear communication while coaching, improved patient communication, and the overall novel approach to teaching.

*“With CET they were very clear and able to tell me what they wanted and communicate with me how they wanted me to do something and what the expectations were.”*

In contrast, the control group asked more knowledge-based questions and usually provided the answer to the questions without providing the opportunity for the learner to process the question and respond.

*“...some other [control residents] were a little more all over the place. You wouldn't know what the expectations from day-to-day or what you are going to get or how things are going to go. If they assign you a topic, you don't know if you are going to discuss it that day, five days later, or never. So that was something I knew with CET, I knew if they assign me something to read, they will ask me the next day and I need to prepare for it so we always had that discussion. I knew what my expectations were … ”*

Through direct and indirect assessments, the most impactful observed change was the strength of association between concepts related to clinical education, especially reflective practice, feedback, mentoring, precepting, and teaching (Fig. 1).

We also assessed the progression of the CET group through focused group discussion with the medical students. In comparison to the control group, medical students reported that CETs were clear and concise in communicating teaching objectives for teaching activities, and professionally conducted the bedside teaching sessions. All medical students concurred that the CET group conducted teaching in a standardized fashion, while some residents in the control group demonstrated similar organization in their teaching, they were not consistent with significant variability between residents.

*“It has created structure in how to approach bedside teaching and improve my knowledge of various forms of bedside teaching, how to effectively give feedback, and how to reflect with more organization.”*

## Discussion

Historically, training programs have relied on residents to teach medical students without equipping the residents with the knowledge and skills to be an effective educator [21]. In this study, the emphasis on communication and professionalism was purposeful. Although the rotation was limited in time to 4 weeks, by the end of their rotation, CETs had successfully achieved the objectives of the rotation. The assessment tools demonstrated that CETs developed a mindful stance and actively practiced reflection, feedback, process observation, precepting and effective questioning techniques.

*“Implementation of a variety of teaching strategies appropriate to learners, engage in critical thinking and create opportunities to do so, using information technology to support the learning process, role model.”*

Triangulation of data sources, such as the direct and indirect methods to collect data from multiple stakeholders, improved the robustness of the study. Furthermore, the CET group demonstrated behavioral changes, that persisted after completion of the rotation, related to communication, professional engagement, practice-based learning, and systems-based learning. Although, our goal is for long-term positive behavioral changes in teaching skills, we are unable to make such a claim beyond the confines of the study period.

Becoming an effective clinical educator is a challenging process that requires time, deliberate effort, and practice [22]. Hence, a transformative approach to medical education is needed to innovate an integrated pedagogical strategy. Gonzalo et al. (2018), outlined some of the gaps in the competencies and curricular domains needed to reform medical education. Their viewpoint is a paradigm shift in how faculty and academic centers should approach healthcare education compared to current practices [16]. In addition, Gonzalo et al., provided a roadmap to transform medical education emphasizing informatics, teamwork, leadership, population health, socio-ecological health from a systems science perspective [23]. Given the rapid changes in healthcare systems, it is imperative for graduate medical education to adapt to the dynamically complex healthcare system and restructure the curriculum to help learners develop the skills needed to become effective educators and leaders [24]. These studies are in line with our research initiative to develop clinical educators early during residency.

Several studies have demonstrated the benefits of developing a clinical educator program [11, 13, 25, 26]. Reported outcomes from these studies were positive for improvement in the level of skills [11], impact on career choices [11,12], development as a clinical educator [12], increasing opportunities to teach [25] and improved feedback skills [26]. Our study identifies similar benefits and further expands on the outcomes of clinical educator programs to include improvement in communication, practice-based learning, engagement and systems-based learning.

Interestingly, the benefits gained from the CET rotation were not confined to the CETs; we observed active transfer of skills from the CETs to other learners, such as morning huddle presentation skill and leading small group activities. Benefits were also noted in the assessments of presentations and huddle by learners, where both the CET and control group showed improvement. We believe this is secondary to reactivity of measurement where participant controls benefit from CETs by being in proximity and observing them during their training [27]. This is a favorable outcome where further dissemination of knowledge and skills occurs among all participants.

### Limitations and challenges

The overarching challenges in this study were primarily related to faculty time and the broad faculty expertise required to manage all the modules coupled with a short rotation schedule. Residents’ self-selection into the test group may have biased the results of the study; however, similar to other ‘elective’ clinical rotations, residents are given the freedom to select what they want to learn in line with their interests. We also noticed some discrepancy in individualized learning challenges stemming from workload and family balance issues. This led to a variability in ability to complete the required readings during the rotation. Another limitation is potential bias by the main faculty member who facilitated the modules in the first 2 weeks covering communication and practice-based learning. However, we sought input from all faculty regarding observed changes in resident behaviors and progress which confirmed our findings. There may be limitations to the generalizability and transferability of the results of this study because the study was conducted at one clinical setting. Participant comments at the end of the study expressed their desire to see the rotation expanded to include all residents. However, anchoring traditional clinical-educator learning using practical exercises that draw from implemented available technologies such as the electronic medical record can be challenging.

## Conclusion

This mixed methods approach used in the study provides evidence that residents can benefit from attending a time-limited rotation that builds on the foundations of teaching and professional growth. This study suggests that to build residents’ teaching ability and proficiency as a clinical educator, exposing them to the theory and practice of topics on practical contemporary education theory, facilitation, and communication skills is an effective strategy. Other benefits besides improving delivery of medical student education may include enhanced patient communication and education, increased resident confidence, personal satisfaction with training, work life-balance, and enhanced career satisfaction. Challenges that may limit such experiences are primarily resource driven especially time and availability of experienced faculty. Future research should focus on curricular content, faculty development and delivery assessment. Also, research efforts should identify appropriate emerging technologies to include in the curriculum for enhancing teaching capacity.

## Data Availability

The datasets used and/or analysed during the current study are available from the corresponding author on reasonable request.

## Abbreviations

CET: Clinical educator trainee
EHR: Electronic health records
GME: Graduate medical education
IM: Internal medicine
PCMH: Patient centered medical home
PGY: Post graduate year
